# Evolution and Progress of mRNA Vaccines in the Treatment of Melanoma: Future Prospects

**DOI:** 10.3390/vaccines11030636

**Published:** 2023-03-13

**Authors:** Dimitrios Bafaloukos, Ioanna Gazouli, Christos Koutserimpas, George Samonis

**Affiliations:** 1 First Department of Medical Oncology, “Metropolitan” Hospital, Neon Faliron, 18547 Attica, Greece; 2 Department of Orthopaedics and Traumatology, “251” Hellenic Air Force General Hospital of Athens, 11525 Athens, Greece; 3 Department of Medicine, University of Crete, 71500 Heraklion, Greece

**Keywords:** mRNA, vaccines, melanoma, cancer, immunotherapy

## Abstract

mRNA vaccines encoding tumor antigens may be able to sensitize the immune system of the host against cancer cells, enhancing antigen presentation and immune response. Since the breakout of the COVID19 pandemic, interest in mRNA vaccines has been accelerating, as vaccination against the virus served as a measure to limit disease spread. Given that immunotherapy has been the cornerstone of melanoma treatment over the last several decades, further innate immunity enhancement by targeted mRNA vaccines could be the next pivotal achievement in melanoma treatment. Preclinical data coming from murine cancer models have already provided evidence of mRNA vaccines’ ability to induce host immune responses against cancer. Moreover, specific immune responses have been observed in melanoma patients receiving mRNA vaccines, while the recent KEYNOTE-942 trial may establish the incorporation of the mRNA-4157/V940 vaccine into the melanoma treatment algorithm, in combination with immune checkpoint inhibition. As the existing data are further tested and reviewed, investigators are already gaining enthusiasm about this novel, promising pathway in cancer therapy.

## 1. Introduction

Since mRNA was discovered and recognized as an indispensable and potent gene transcription mediator [1], artificially inducing protein expression into cell cultures and murine models has been extensively applied in cancer research [2,3,4]. During the 1990–2000 decade, several attempts at mRNA-based anti-cancer vaccine development were made on a preclinical level, using induced expression of established cancer antigens such as carcinoembryonic antigen (CEA) and glycoprotein 100 (gp100). [5,6,7].

Nonetheless, mRNA-based vaccines had not been largely incorporated in clinical practice until the outburst of the COVID19 pandemic, mainly due to the lack of adequate scientific and technical means to reassure their immunogenic effect as well as their stability [8,9]. Over the last decades, expertise regarding mRNA vaccine production gradually increased, eventually allowing them to become the primary protection milestone against the recent pandemic [8,9]. Indeed, through the years 2020–2021, Pfizer and Moderna mRNA vaccines were studied in clinical trials, were officially approved, and were administered to the public in an effort to restrict the spread of the virus and decrease the severity of its clinical manifestations in infected individuals [10,11,12]. In this context, scientific interest in mRNA vaccines as an antineoplastic treatment has been revived.

mRNA vaccines mediate antigen presentation, as they are incorporated by dendritic cells, which consequently express the vaccine-encoded cancer antigens on their surface, thus inducing cytotoxic CD8+ as well as helper CD4+ cell activation while increasing the release of inflammatory mediators [13]. Hence, they represent a promising way of delivering genetic information to immune cells without interfering with nuclear DNA structure or affecting cellular protein expression in a permanent manner, as mRNA does not penetrate the cell nucleus, which might induce hazardous mutations [8,9,14,15]. In addition, mRNA may be transferred without viral or plasmid vectors, is naturally dissolved by the host cell, and it is less costly to produce compared to DNA-related therapeutics, enabling even safer administration and large-scale production [8,9,13,16,17].

mRNA vaccines may be administered ex vivo; antigen-presenting cells—such as dendritic cells—are isolated from the patient, incubated with the mRNA vaccine in order to induce expression of mRNA-encoded antigens, and finally re-introduced to the host. An alternative approach consists of direct administration of the mRNA vaccine to the patient, and requires a secured vaccine structure, made feasible by integration of stabilizing cationic complexes such as protamine (a resin-like alkaline protein) and polymers such as polyethylenimine [8,9,18]. More recent technological advances have led to the development of lipid nanoparticles used as mRNA vectors, safely transporting mRNA into the cytoplasm, as they are both stable and prone to endocytosis, without interfering with the loaded mRNA function [8,9,18].

During recent decades, the process of mRNA vaccine production has been extensively investigated and refined. Transcription of the mRNA molecule involved is performed in vitro, based on the DNA sequence encoding the targeted antigen, the latter being incorporated in a linearized plasmid [8,9,18]. Having escaped degradation by extracellular RNases (enzymes decomposing mRNA), a fraction of the administered mRNA enters the cytoplasm of the targeted cell by endocytosis, to be translated into proteins by the ribosomal machinery. The resulting protein may be either extracellularly released, or transported and exposed on the extracellular surface, attached on MHC (major histocompatibility complex) class I or II proteins [8,9,18].

As already mentioned, stability is key to efficient mRNA vaccination, given the frail nature of mRNA and the vast presence of extracellular RNases. Creating a robust mRNA vaccine can be achieved by incorporation of 5′ and 3′ untranslated regions, which encompass the encoding area, preventing its degradation. Capping by methylation of the 5′ area, and attachment of a poly(A) tail (a sequence of multiple adenosine monophosphates) to the 3′ area, are both employed to further stabilize the mRNA sequence [8,9,18].

mRNA-based treatments have wide-ranging potential; they may be applied against malignancies, infectious diseases, and allergies. In oncology, the goal of mRNA vaccination, regardless of the administration method or the encoded sequences, is to amplify immune surveillance and reinforce host immune system activity against cancer cells [8,9,16,18].

Target proteins encoded by mRNA vaccine sequences investigated in the field of oncology belong to one of three main categories: 1. Neoantigens, or mutated protein forms exclusively expressed by the tumor, due to DNA alterations, alternative mRNA splicing, or post-transcriptional changes. They are characterized by high and tumor-specific immunogenicity and may be associated with tumor type or even be personalized, patient-specific antigens [19]. 2. Tumor-associated antigens, which may be found on normal tissue, their expression deviating quantitatively or structurally from normal patterns, such as MAGE-A3 (MAGE family number A3), NY-ESO-1 (New York esophageal squamous cell carcinoma 1), tyrosinase, TPTE (transmembrane phosphatase with tensin homology), and gp100 [20]. 3. Inflammatory mediators, either chemokines extracellularly excreted such as IL-12 (interleukin-12) and GM-CSF (granulocyte-macrophage colony-stimulating factor), or expressed on cellular surface such as TLR4 (toll-like receptor 4) [21]. Isolation of the above proteins and mRNA sequences, and recognition of the most immunogenic neoantigens and the corresponding DNA alterations, has allowed creation of suitable DNA templates that may be employed in the production of various mRNA vaccines, which may be applied to various types of malignancies [8,9,16,18,21].

Immune checkpoint inhibitors are monoclonal antibodies targeting specific receptors on the surface of immune or malignant cells, disabling deactivation of host cytotoxic T lymphocytes that may be induced by the tumor cells. Such agents (e.g., pembrolizumab, nivolumab, ipilimumab) have revolutionized cancer therapeutics since 2010, boosting treatment probabilities for various neoplasms, inducing durable objective responses, and significantly prolonging patient survival [22]. More importantly, immunotherapy became the principal treatment for patients with non-chemosensitive neoplasms such as melanoma, providing tolerable and effective treatment options [22]. However, immune escape may still occur; as shown by clinical trial data, 50% and 64% of melanoma patients, even under the potent combination of ipilimumab and nivolumab, will experience disease progression at 1 and at 5 years after treatment initiation, respectively [23].

Underlying resistance mechanisms to immunotherapy may be summarized as follows:

1. Reduced expression of target molecules, such as PD-L1 (programmed death-ligand 1) by cancer cells, interfering with the effectiveness of anti-PD-1 (programmed cell death protein 1) antibodies. Anti-PD-1 agents, such as nivolumab and pembrolizumab, are designed to inhibit the immunosuppressive interaction between immune and malignant cells, which is mediated by linkage between PD-1, expressed by T lymphocytes, and PD-L1, expressed by malignant cells. Consequently, low PD-L1 expression has been considered to indicate primary resistance [24,25].

2. Low neoantigen burden of malignant cells. Neoantigens are specific neoplastic antigens, deriving from genetic alterations carried by the tumor; the higher the tumor mutational load, the greater the variety of altered antigens presented on the cancer cell surface. These modified cancer antigens are recognized as foreign by the host immune system, enhancing immune infiltration and cytotoxicity. Tumors carrying limited neoantigens may surpass immune surveillance, and be less responsive to immune checkpoint inhibitors [26,27].

3. Immunosuppression. It has been found that cancer cells, but also myeloid-derived cells, tumor stroma cells, and CD4+ regulatory T lymphocytes, may lead to immune cell inactivation by promoting excretion of suppressive cytokines such as IL-10 (interleukin-10) and other chemical mediators such as TGF-beta (tumor growth factor-beta), which inhibit immune cell infiltration and amplification, and production of inflammatory cytokines [28,29,30].

**Figure 1:**
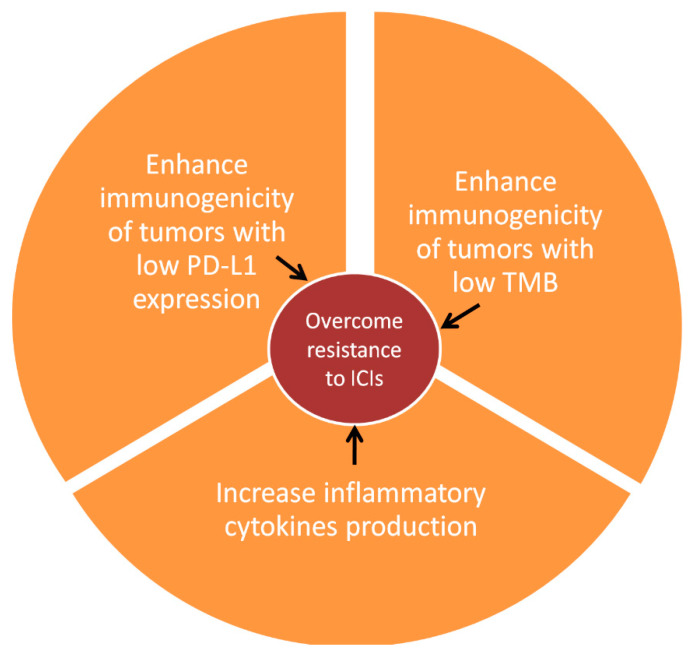
Schematic representation of mRNA vaccine interaction with the immune system, aiming to enhance immune checkpoint immunotherapy. TMB: Tumor mutational burden, ICIs: Immune checkpoint inhibitors.

In this context, mRNA vaccination aspires to become a valuable complement to immune checkpoint inhibitors, reversing resistance pathways (Figure 1). Antigens crucial to immune system stimulation (including both patient- or tumor-type-specific neoantigens and tumor-associated antigens) encoded by mRNA vaccines can be expressed on the cellular surface of antigen-presenting cells, facilitating recognition of tumor nests by the host immune system, regardless of the innate tumor neoantigen production or PD-L1 expression [8,9,16]. In parallel, mRNA vaccines encoding immune-activation-associated molecules, such as IL-12, IFNα (interferon-alpha), GM-CSF, and TLR4, might be able to counter-balance cancer-cell-induced immune suppression by restoring immune cell activity and inflammatory mediator production [8,9,16]. Indeed, in a recently published experiment, an mRNA vaccine encoding for single-chain IL-12 (fusion of the IL-12p40 and IL12p35 subunits), IFN-α, GM-CSF, and IL-15-sushi (fusion of IL-15 to the sushi domain of the IL-15 receptor), managed to overcome resistance to anti-PD-1 treatment in a colon adenocarcinoma murine model, inducing tumor shrinkage and prolonging survival of the treated mice [31]. Subsequently, co-administration of mRNA vaccines and immune checkpoint inhibitors has become an intriguing future therapeutic strategy [8,9,16].

Targeted immunotherapy has been successfully applied to melanoma, a neoplasm with a well-established relationship with the immune system [32]. However, metastatic melanoma remains a deadly disease for a significant portion of patients, imposing the need for further research for a decisive treatment. Immunogenic mRNA vaccination may become the next substantial step towards this goal. In the present review, we attempt to describe the recent preclinical and clinical data regarding mRNA vaccines in melanoma treatment, as well as future prospects and potential applications.

## 2. Preclinical Evidence

mRNA vaccines have been evaluated in preclinical murine cancer models in various experiments (Table 1). Stabilization by lipid calcium phosphate nanoparticles (LCPs) has been shown to improve efficiency of an mRNA vaccine encoding gp100 and tyrosinase-related protein 2 (TRP-2) that was administered to immunocompetent murine B16F10 melanoma models. Vaccination induced significant tumor shrinkage, while prolonging survival of the treated mice [33].

In 2018, Wang et al. [34] reported successful in vitro transfection of dendritic cells by an LCP-based vaccine containing mRNA encoding tyrosinase-related protein 2 (TRP-2) and silencing RNA (siRNA) targeting PD-L1 expression. TRP-2 is a protein mediating melanin synthesis in melanocytes, and has been reported to confer melanoma cell resistance against DNA-damaging agents when overexpressed [38]. When the murine melanoma models were directly vaccinated, CD8+ T lymphocyte generation in lymph nodes, tumor mass, and spleen was increased compared to untreated animals. T lymphocyte-specific reaction to TRP-2 was enhanced, while PD-L1 (programmed death- ligand 1) expression was effectively knocked down. Tumor growth was significantly delayed in treated animals, as well as growth of cancerous lymph nodes. Interestingly, vaccination by combination of TRP-2-encoding mRNA and siRNA was found to more prominently delay tumor growth compared to co-administration of a TRP-2 mRNA vaccine and an anti-PD-1 (programmed cell death protein 1) monoclonal antibody. It was also noted that LCP formations seemed to promote dendritic cell maturation, via enhanced intracellular calcium release.

Another LCP-based mRNA vaccine, also encoding TRP-2, managed to infiltrate antigen-presenting cells (APCs) by phagocytosis, inducing vigorous T cell activation, and promoting toll-like receptor 4 (TLR4)-mediated signaling and inflammatory cytokine release when subcutaneously injected in melanoma murine models. As a result, tumor growth was considerably delayed in vaccine-treated mice in comparison to untreated controls [35]. Intratumoral injection of a combined vaccine containing synthetic phosphorothioate-modified CpG oligodeoxynucleotides (CpG-ODNs) has been shown to enhance immune response and mRNA encoding for specific melanoma neoantigens in syngeneic murine models, inhibiting melanoma growth while promoting tumor immune infiltration by CD4+ and CD8+ lymphocytes [36]. In a recently published experiment, mRNA encoding TRP-2 and ovalbumin, an egg white protein shown to enhance neoantigen recognition by cytotoxic lymphocytes [39], delivered to lymph nodes of syngeneic melanoma murine models, managed to promote a cytotoxic cell response by CD8+ T cells. In parallel, in combination with an anti-PD-1 inhibitor, complete responses were observed in 40% of the treated mice. Vaccination has been shown to result in long-term immune memory in rechallenge attempts, where metastatic tumor growth was inhibited in vaccinated animals [37].

## 3. Clinical Evidence

mRNA vaccines have been administered to advanced melanoma patients in the context of several phase I/II clinical trials (Table 2). As early as 2006, a vaccine consisting of autologous monocyte-derived dendritic cells, ex vivo loaded with autologous tumor mRNA, has been intranodally or intradermally injected to 22 malignant melanoma patients. Vaccine-specific immune reaction, characterized by T lymphocyte expansion and interferon-γ production was, indeed, observed in nine out of 19 patients, evaluated by T cell proliferation/interferon-*γ* ELISPOT assays, as well as in 8/18 evaluable by delayed hypersensitivity reaction [40]. Intradermal or intranodal administration induced immune response in 70% (7/10) and in 25% (3/12) of treated patients, respectively [40]. Later on [41], immune-specific CD4+ and CD8+ T cell responses against neoantigens encoded by the vaccine mRNA were reported among nine of the responders; patients’ T cells isolated post-vaccination were able to produce various T cell clones specifically reacting to dendritic cells, while a broad variety of T cell receptors reflecting the vaccine neoantigen spectrum were noted.

Direct intradermal administration of protamine-stabilized mRNA encoding for melanoma antigens (Melan-A, Tyrosinase, gp100, MAGE-A1, MAGE-A3, Survivin) to 21 metastatic melanoma patients [42] was well-tolerated, inducing no adverse events of grade 3 or higher. Markedly, regulatory and myeloid suppressor cell circulation was limited in vaccinated patients. Specific T lymphocyte immune reaction against vaccine antigens was noted in two out of four evaluable patients, and a complete response was observed in one out of seven patients with measurable disease.

The TriMix mRNA vaccine, consisting of mRNA encoding for CD40 ligand (T helper cell surface protein, mediating antigen-specific reaction), constitutively active toll-like receptor 4 (hematopoietic and nonhematopoietic cell surface antigen mediating recognition of exogenous and endogenous antigens), and CD70 (tumor immune checkpoint antigen), has been tested in various trials. In a pilot study [43], autologous TriMix-electroporated dendritic cells were transfected by mRNA encoding for melanoma-associated antigen (MAGE-A3, MAGE-C2, tyrosinase, or gp100), conjugated with an HLA class II signal. Transfected dendritic cells were safely administered to 35 stage III/IV inoperable melanoma patients, but no objective responses according to RECIST criteria were observed. After additional IFN-α-2b administration, 1/17 evaluable patients experienced a partial response, while 5/17 presented stable disease. Skin biopsies performed in 21 patients after a fourth TriMix-DCs injection showed infiltration by vaccinal neoantigen-specific T lymphocytes in 12 of them. Notably, autologous vaccination with TriMix-DCs thawed with melanoma-associated antigens (MAGE-A3, MAGE-C2, tyrosinase, gp100) has been shown to induce expansion of vaccinal neoantigen-directed T lymphocytes, found present both in peripheral blood samples in 11/14 and in skin biopsies in 12/14 of evaluable treated patients [44]. Among 14 evaluable patients, two complete and one partial objective response were noted, with another 4/14 patients showing disease stabilization. PFS and OS varied from 1.8 to 51 months, and 6.4 to 51 months, respectively; but no robust association between clinical outcome and immunological response was observed in the study [44]. TriMix vaccination has been reported to induce evaluable immune responses in 4/10 patients with advanced stage melanoma receiving a high dose regimen, and in 3/9 patients receiving a low dose regimen, in ASCO 2019 [47].

The same TriMix-DC-MEL vaccine, based on mRNA coding for four melanoma-associated antigens (tyrosinase, gp100, MAGE-A3, and MAGE-C2), has been also combined with ipilimumab administration in 30 advanced melanoma patients [46]. Reported five-year overall and progression-free survival rates were 28% and 18% respectively. Immune response assessment by peripheral blood mononuclear cell (PBMC) retrieval and evaluation for melanoma vaccinal antigen enrichment was feasible for 15/30 patients (4/15, 4/15 and 2/15 with CR, PR and SD, respectively). Immune response to the vaccine was noted in 12/15 patients and was significantly associated with clinical objective responses, being more robust in patients with partial and complete responses compared to patients with stable or progressive disease. Notably, overall survival was found to be related with the percentage of CD8+ T cell activation in immune responders [46].

Vaccination with autologous dendritic cells loaded with mRNA encoding for melanoma-specific antigens (MAGE-A1, -A3, -C2, tyrosinase, MelanA/MART-1, or gp100), and an HLA class II-targeting sequence, has also been evaluated in 30 resected stage III/IV melanoma patients [45]. Reported median relapse-free survival was almost two years (22 months; 95% CI 12–32 months). By the time of publication, twelve patients were deceased, and four-year overall survival rate was 70%. Median overall survival was not reached.

In an attempt to broaden the melanoma-associated neoantigen spectrum applied to mRNA vaccines, Ping et al. [48] compared 471 melanoma tissue samples to 812 normal skin samples. A total of five potentially targetable tumor antigens were identified (PTPRC, SIGLEC10, CARD11, LILRB1, ADAMDEC1); high antigen expression was associated with prolonged OS and DFS, as well as higher tumor infiltration by antigen-presenting cells. Robust expression of these five antigens by the cancer cells was associated with more robust tumor immune infiltration and improved patient overall survival, whereas lower expression levels and shorter survival time were associated with immunogenically ‘cold’ melanomas. Such observational studies could contribute to the recognition of highly immunogenic antigens, which could serve as a basis for novel mRNA vaccine construction.

Currently, mRNA vaccination is being evaluated in six melanoma clinical trials, which are already exhibiting promising results [49,50].

KEYNOTE-942 (NCT03897881) [33,34], an ongoing open-label phase IIb trial, has already shown particularly encouraging results regarding the adjuvant treatment setting. In this study, a combination of a personalized mRNA vaccine encoding 20 different mutated neoantigens and the anti-PD-1 inhibitor pembrolizumab has been administered to patients with completely resected stage III/IV melanoma, compared to single-agent pembrolizumab treatment. According to a recent press release by the producer company [51], patients receiving adjuvant treatment with pembrolizumab combined with the mRNA-4157/V940 vaccine seem to have a 44% lower risk of disease relapse or death, compared to patients under single-agent pembrolizumab treatment (HR = 0.56, 95% CI, 0.31–1.08; one-sided *p*-value = 0.0266). Severe treatment-related adverse events were reported at a rate of 14.4% and 10%, in the combination and single-agent pembrolizumab arm, respectively [51].

Reflecting the investigators’ enthusiasm about the KEYNOTE-942 early outcomes, Professor Georgina Long of the Melanoma Institute of Australia stated that this trial may generate a “penicillin moment” in regard to melanoma therapy [52]. Furthermore, a phase III trial is also being planned in order to be initiated as the next step [53].

Safety and tolerability of the BNT111 mRNA vaccine, which encodes four melanoma antigens: NY-ESO-1, tyrosinase, MAGE-A3, and TPTE, is under evaluation in a phase I trial (NCT02410733). This same vaccine is being co-administered with another PD-1 inhibitor, cemiplimab, in a three-arm comparative phase II trial (NCT04526899) [50]. Researchers will be attempting to compare monotherapy with BNT111 vaccine or cemiplimab to the combination of both agents, as second-line treatment against immune checkpoint inhibitor-refractory, unresectable melanoma [50].

A Memorial Sloan Kettering Cancer Center phase I trial is currently evaluating administration of autologous human Langerhans-type dendritic cells, electroporated with an mRNA vaccine encoding for TRP-2, to patients with IIB to IV stage melanoma, after appropriate surgical treatment (NCT01456104) [50]. An autologous mRNA vaccine based on gp100, tyrosinase, PRAME, MAGE-A3, IDO, and other tumor driver mutations, loaded on dendritic cells, is to be administered in uveal melanoma patients, in combination with conventional treatment, in a phase I trial (NCT04335890) [33]. Moreover, a phase I open-label trial (NCT05264974) [50], scheduled to initiate patient recruitment in 2023, aims to explore tolerability of an autologous tumor mRNA nanoparticle vaccine in stage IIIB to stage IV melanoma patients, after disease relapse despite adjuvant immunotherapy. The study is expected to be completed in 2027 [50].

## 4. Conclusions and Future Prospects

During the COVID19 pandemic, mRNA vaccines were rigorously studied, revealing the potential of this state-of-the-art technology to innovate melanoma treatment. Animal model experiments and clinical trials have shown promising results, setting a solid background for more systematic research in the years to come. The KEYNOTE-942 trial especially, combining an mRNA vaccine with an immune checkpoint inhibitor, aspires to achieve the next pivotal breakthrough in melanoma treatment.

Important endpoints for future laboratory and clinical research investigating mRNA-based cancer therapeutics would ideally include:

1. Identification of highly immunogenic proteins, either chemokines or tumor-related antigens, which will allow more effective and specific immune system stimulation, without affecting normal cells.

2. Development of expertise regarding knowledge and infrastructure to produce more stable mRNA vaccines that are able to escape early degradation, be safely administered, be produced in a timely fashion, and be distributed in large-scale.

3. Tolerability of the possible combinations of mRNA vaccination with immune checkpoint inhibitors and even chemotherapy or radiotherapy; but also their effectiveness in terms of objective response, disease relapse or progression prevention, overall survival, and quality of life for patients.

4. Clinical benefit of mRNA-based vaccinations in the metastatic, adjuvant, and neoadjuvant treatment setting, as well as in first- or second-line treatment and beyond.

As the above queries remain to be answered, practicing physicians should be aware of recruiting studies in order to inform patients, offering them the opportunity of being enrolled and receiving current technology-based medications.

## Figures and Tables

**Table 1:** mRNA vaccines as treatment of melanoma: Preclinical data.

Experiment Subject	Vaccine Composition	Vaccine Transport	Results	Reference
Aggressive B16F10 murine melanoma models	Lipid nanoparticles-mRNA encoding gp100, TRP-2	Direct vaccine administration	Tumor shrinkage Prolonged overall survival of the treated mice	Oberli et al., 2017 [33]
Immune-competent murine B16F10 melanoma model	LCP-based vaccine mRNA encoding TRP-2 siRNA targeting PD-L1	Transfected DCs transported to mice	Efficient mRNA delivery to DCs in lymph nodes T cell specific reaction to TRP-2 Reduced tumor growth Enhanced CD8+ T cell proliferation	Wang et al., 2018 [34]
Murine melanoma models	Nanovaccine with C1 lipid nanoparticle mRNA encoding TRP-2	Vaccine enters APCs via phagocytosis	TLR4 activation-Robust T cell activation Inflammatory cytokines inductionReduced tumor growth	Zhang et al., 2021 [35]
Syngeneic murine models	Tumor neoantigen mRNA, encapsulated in lipid nanoparticles	Intratumoral vaccine administration	Melanoma growth inhibition Immunogenically ”cold” tumors turn into “hot”	Li et al., 2021 [36]
B16F10 melanoma murine models	Lymph node-targeting lipid nanoparticle with mRNA encoding for ovalbumin, TRP-2	Targeted delivery of mRNA to lymph nodes	Increased CD8+ T cell response Long term immune memory	Chen et al., 2022 [37]

LCP: lipid calcium phosphate nanoparticles, TRP-2: tyrosinase-related protein 2, PD-L1: programmed death ligand 1, DCs: dendritic cells, APC: antigen-presenting cells, TLR4: toll-like receptor 4.

**Table 2:** mRNA vaccines in the treatment of melanoma: Clinical data.

Patient Population	Vaccine-Encoded Antigens	Outcomes	Reference
22 patients with advanced malignant melanoma	Autologous tumor mRNA	Vaccine-specific immune response in 9/19 patients evaluable by T cell assays and in 8/18 patients evaluable by delayed-type hypersensitivity reaction	Kyte et al. 2006 [40]
21 metastatic melanoma patients	Melan-A, Tyrosinase, gp100, MAGE-A1, MAGE-A3, Survivin	Safe, tolerable Antigen-specific T cell reaction in 2/4 patients CR in 1/7 patients	Weide et al. 2009 [42]
35 advanced melanoma patients	Tyrosinase, gp100, MAGE-A3, MAGE-C2	In patients treated by autologous DCs electroporated with mRNA vaccine plus IFN-α-2b: PR:1/17 SD: 5/17	Wilgenhof et al. 2011 [43]
14 recurrent melanoma patients	CD40L, TLR4, CD70 plus tyrosinase or MAGE-A3 or MAGE-C2 or gp100	T cell-specific reaction in 11/14 patients (peripheral blood) and in 12/14 patients (tissue) CR: 2/14 PR: 1/14 SD: 4/14	Benteyn et al. 2013 [44]
30 patients with resected melanoma	Autologous mRNA	mRFS: 22 months (95% CI 12–32 months) 4yr OS 70%	Wilgenhof et al. 2015 [45]
39 advanced melanoma patients	Tyrosinase, gp100, MAGE-A3, MAGE-C2	6mo DCR 51% CR: 20.5% PR: 17.9% T cell stimulation in 12/15 evaluable patients T cell response related to objective response	De Keersmaecker et al. 2020 [46]
157 patients with resected melanoma	20 tumor neoantigens	Decreased risk of relapse/death by 44% compared to pembrolizumab monotherapy	KEYNOTE-942, press release 2022

CR: complete response, PR: partial response, SD: stable disease, gp100: glycoprotein 100, TLR4: toll-like receptor 4, DCR: disease control rate, mRFS: median recurrence free survival, OS: overall survival.

## Data Availability

Not applicable.
